# From a young BSJ member: advanced technologies encouraged me to dive into biophysics field

**DOI:** 10.1007/s12551-020-00648-x

**Published:** 2020-02-23

**Authors:** Kazuko Okamoto

**Affiliations:** Laboratory for Comprehensive Bioimaging, Center for Biosystems Dynamics Research, RIKEN, 3-10-23 Kagamiyama, The Research site in Hiroshima University, Higashi-Hiroshima, Hiroshima, Japan

I am Kazuko Okamoto working as a researcher in RIKEN-Hiroshima University Collaboration Research Facility, which was opened in 2018. In the past 4 years, I have been working for single molecular imaging of transcription factors in living nuclei in order to understand the relationships between chromatin architecture and molecular dynamics of transcription factors. I have been fascinated by microscopy technology developing in biophysics field, including single molecular imaging; therefore, I started to study about the dynamics of transcription factors using such microscopes. Then, I joined The Biophysical Society of Japan (BSJ) 3 years ago, and they gave me a chance to write this commentary here as a young BSJ member.

I studied developmental biology in the early days of my scientific career; thus, I was estranged in the techniques and studies in the biophysics field. I have never thought that it is now possible to observe nucleoproteins including transcription factors at single molecular level and obtain spatio-temporal information inside living nuclei in the past.

The first trigger to get curious about the biological meaning of the relationship between chromatin architecture and transcription regulation was a CCCTC-binding factor (CTCF) protein which plays a role in the regulation of 3D architecture of chromatin and facilitates transcriptional insulation activity (Arzate-Mejía et al. 2018). When I was an undergraduate student, I used sea urchins which are one of the common model organisms in the developmental biology field. I had observed fertilized sea urchin eggs with fluorescence-labeled CTCF in order to understand the relationships between transcriptional regulation and CTCF. At that time, my observation was just an analysis of the expression patterns during embryogenesis, and I had not reached the detailed behavior of CTCF inside living nuclei.

More than 10 years have passed since then; sequencing technology has greatly developed to detect the 3D chromatin architectures (Ohno et al. 2019), and single molecular imaging is now available inside living nuclei (Tokunaga et al. 2008, Coleman et al. 2015). My motivation to study the biological meaning of the relationship between chromatin architecture and transcription regulation revives, and I face the issue again.

**Fig. 1:**
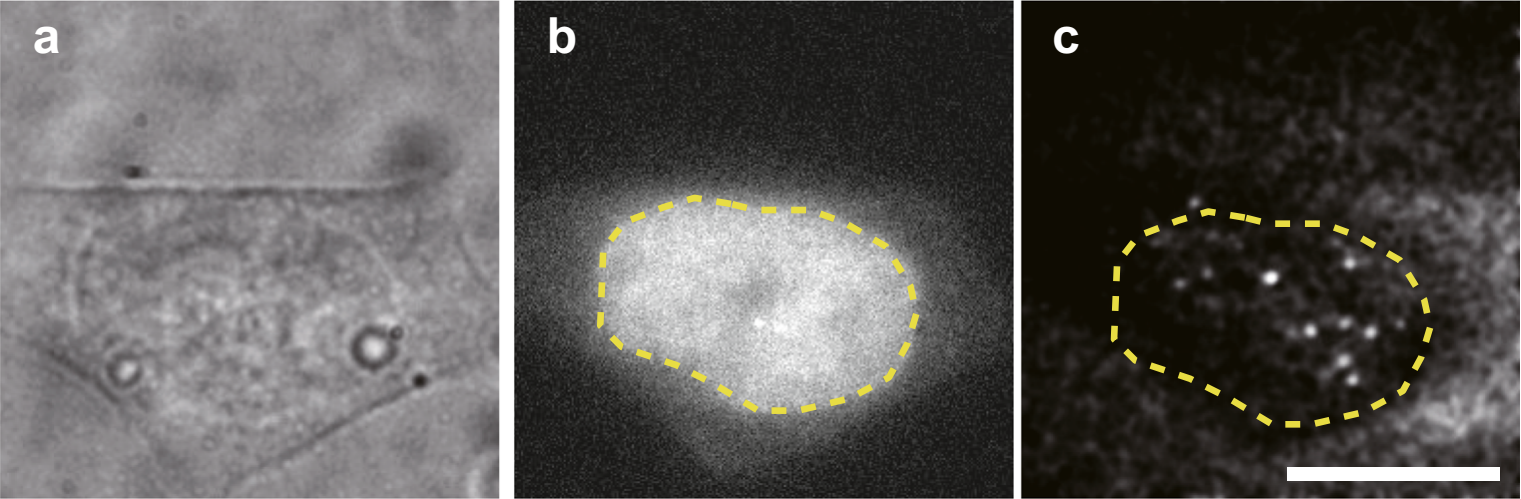
Example images of single molecular imaging of transcriptional proteins. **a** Bright-field image. **b** Epi-illumination of GFP-fused transcriptional proteins. **c** Single molecular image of GFP-fused transcriptional proteins. White dots indicate single molecules of transcriptional proteins. Scale bar, 5 μm. Yellow dashed line indicates the nuclear membrane

My question is very simple. Chromatin architectures control the transcription process by the binding of core transcriptional proteins, and then cellular states change. However, it is poorly understood how the chromatin architecture controls the binding of core transcriptional proteins. Now, I am enthusiastically involved in the observation of single molecular behavior of transcriptional proteins (Fig. 1).

In my case, advanced microscopic technology encouraged me to join biophysics field, and I am trying to understand the mechanisms of the interplay between chromatin architecture and transcription regulation again. The studies in transcription process will gain more attention in biophysical studies in Japan, and I am willing to contribute to the understanding of the transcription machinery by observing molecular behaviors of transcriptional proteins one by one. Finally, I would like to thank the BSJ for giving me the Early Career Presentation Award at the 57th Annual Meeting of BSJ in Miyazaki.
